# Monthly Summary

**Published:** 1887-08

**Authors:** 


### Monthly Summary.

TEMPERATURE OF THE HUMAN SKIN.—Jointly with the Messrs. Kellermann and Wiedemann, Mr. Kunkel has endeavored to ascertain the temperature of the exterior teguments. For this purpose, he availed himself of a thermoelement of German silver and iron, in the shape of a seal, and of easy manipulation (as described more accurately in the original communication). One single determination (until the needle of the galvano-meter has reached its definite position of rest) required twelve seconds only. The apparatus, although not entirely free of occasional mistakes of observation, gives results which may be called highly satisfactory. In men from 20 to 30 years of age, temperatures were found as follows. Skin of the face about 31° C, (between 29.5 and 32); protruding parts of the head, such as the tip of the nose, lobes of the ear, showed temperatures as low as 24° C., and even 1-2° less, not unfrequently; hand 27-29°. Besides this, the skin in places where muscles are located, shows itself to be hotter in comparison with places over bones and tendons. The difference is 1° and more. Muscular contractions increases the temperature of the skin to the amount of 0.6°. As the contraction ceases, the small increase in temperature gradually recedes. For the trunk, 30-32° were found, also in places which are covered with clothes; for the skin on the foot 26.5-28.0°. At 1 F. 5° C. temperature of the room, the temperature was:

| | |
|---|---|
| On the coat | 22.3° C. |
| " waistcoat | 24.2 |
| " linen shirt | 28.2 |
| " skin | 31.2 |

At 19.5° temperature outside:

| | |
|---|---|
| On the worsted coat | .25-3° C. |
| " linen shirt | 27.8 |
| " woolen shirt | 28.9 |
| " free surface of the skin | 31.4 |

Lastly, at 21° temperature of the room, over the region of the liver:

| | |
|---|---|
| On the waistcoat | 23.7° C. |
| " linen shirt | 23.7 |
| " woolen shirt | 27.3 |
| " skin | 31.5 |

The highest temperature was shown generally by the vigorous age. At 2 years old a child showed from 25-28° temperature of the skin (in lightly covered places from 26-27°). Two 14-year old boys showed 27-29°.—*Reports of the Meetings of the Physikalisohmedizinisohe Gesellshaft zum Wurzburg*, 1886.

